# Association of single nucleotide polymorphisms in FOXE1 and pre-MIR146A with papillary thyroid carcinoma

**DOI:** 10.1186/1471-2164-15-S2-P68

**Published:** 2014-04-02

**Authors:** Nadia Bagatian, Ohoud Subhi, Shireen Hussain, Khalid Al-Ghamdi, Osman Abdel Al-Hamour, Mohammed Hussein Al-Qahtani, Adeel Chaudhary, Adel Abuzenadah, Jaudah Al-Maghrabi, Hans-Juergen Schulten

**Affiliations:** 1 Center of Excellence in Genomic Medicine Research, King Abdulaziz University, Jeddah, Kingdom of Saudi Arabia; 2 Department of Surgery, Faculty of Medicine, King Abdulaziz University, Jeddah, Kingdom of Saudi Arabia; 3 Department of Surgery, King Faisal Specialist Hospital and Research Center, Jeddah, Kingdom of Saudi Arabia; 4 Department of Pathology, Faculty of Medicine, King Abdulaziz University, Jeddah, Kingdom of Saudi Arabia

## Background

Papillary thyroid carcinoma (PTC) is one of the most common cancer types in the Middle East and North African (MENA) region and more abundant than in many other world regions suggesting that genetic susceptibility factors for this malignancy are likely to vary between the populations studied. We assessed in a population from the MENA region the allele frequencies of two SNPs which may bear the capacity to confer risk for developing PTC. SNP rs2910164 is a sequence polymorphism in the precursor microRNA (mir)146a. The heterozygous C/G state of rs2910164 was found to be associated with a reduced amount of mir146a which in turn had an effect on the efficiency to inhibit target genes as CCDC6 [1,2]. SNP rs1867277 is located 283 bp upstream of the translational start site of the developmental gene encoding forkhead box E1 (FOXE1) and identified as an associated risk factors for a number of solid tumors including PTC [3,4].

## Materials and methods

SNPs rs2910164 and rs1867277 were investigated in patients with PTC and volunteers without a thyroid disease (case and control group each, N=207-234) using PCR, including a multiplex PCR step on genomic DNA, to amplify each SNP region. PCR products were directly sequenced. For Hardy-Weinberg (HW) equilibrium testing the Online Encyclopedia for Genetic Epidemiology studies (http://www.oege.org) was used. Odds ratios (ORs) and 95% confidence intervals (CI) were calculated using the online VassarStats website for statistical computation (http://vassarstats.net/index.html) and considered to be statistically significant for a P-value ˂ 0.05.

## Results

Both SNPs rs2910164 (mir146a) and rs1867277 (FOXE1) were in HW disequilibrium in the patient group (*X*^2^ = 8.33 and *X*^2^ = 8.29, respectively) but in equilibrium in the control group (*X*^2^ = 0.87 and *X*^2^ = 1.56, respectively). For rs2910164 a trend association with PTC was found for the heterozygous C/G state when compared to the combined C/C+G/G states (OR = 0.51, CI 0.51-1.1, P = 0.11). For rs1867277 the risk allele A was significantly associated with PTC in comparison to allele G (OR = 1.94, CI 1.48-2.53, P < 0.0001).

## Conclusions

The HW disequilibrium of SNP rs1867277 in the PTC group and its significant association of risk allele A with PTC let us suggest that this SNP in the developmental FOXE1 gene may represent an additive risk factor for developing PTC in the investigated population.

*This study was supported by King Abdulaziz City for Science and Technology* (*KACST*) *grants 09-BIO707-03 and 09-BIO820-03*.
